# ‘They care rudely!’: resourcing and relational health system factors that influence retention in care for people living with HIV in Zambia

**DOI:** 10.1136/bmjgh-2018-001007

**Published:** 2018-10-25

**Authors:** Chanda Mwamba, Anjali Sharma, Njekwa Mukamba, Laura Beres, Elvin Geng, Charles B Holmes, Izukanji Sikazwe, Stephanie M Topp

**Affiliations:** 1 Centre for Infectious Disease Research in Zambia, Lusaka, Zambia; 2 Bloomberg School of Public Health, Johns Hopkins University, Baltimore, Maryland, USA; 3 School of Medicine, University of California, San Francisco, California, USA; 4 College of Public Health Medical and Veterinary Sciences, James Cook University, Townsville, Queensland, Australia

**Keywords:** HIV, health system, service delivery model, retention in care, loss to follow up

## Abstract

**Introduction:**

Despite access to free antiretroviral therapy (ART), many HIV-positive Zambians disengage from HIV care. We sought to understand how Zambian health system ‘hardware’ (tangible components) and ‘software’ (work practices and behaviour) influenced decisions to disengage from care among ‘lost-to-follow-up’ patients traced by a larger study on their current health status.

**Methods:**

We purposively selected 12 facilities, from 4 provinces. Indepth interviews were conducted with 69 patients across four categories: engaged in HIV care, disengaged from care, transferred to another facility and next of kin if deceased. We also conducted 24 focus group discussions with 158 lay and professional healthcare workers (HCWs). These data were triangulated against two consecutive days of observation conducted in each facility. We conducted iterative multilevel analysis using inductive and deductive reasoning.

**Results:**

Health system ‘hardware’ factors influencing patients’ disengagement included inadequate infrastructure to protect privacy; distance to health facilities which costs patients time and money; and chronic understaffing which increased wait times. Health system ‘software’ factors related to HCWs’ work practices and clinical decisions, including delayed opening times, file mismanagement, drug rationing and inflexibility in visit schedules, increased wait times, number of clinic visits, and frustrated access to care. While patients considered HCWs as ‘mentors’ and trusted sources of information, many also described them as rude, tardy, careless with details and confidentiality, and favouring relatives. Nonetheless, unlike previously reported, many patients preferred ART over alternative treatment (eg, traditional medicine) for its perceived efficacy, cost-free availability and accompanying clinical monitoring.

**Conclusion:**

Findings demonstrate the dynamic effect of health system ‘hardware’ and ‘software’ factors on decisions to disengage. Our findings suggest a need for improved: physical resourcing and structuring of HIV services, preservice and inservice HCWs and management training and mentorship programmes to encourage HCWs to provide ‘patient-centered’ care and exercise ‘flexibility’ to meet patients’ varying needs and circumstances.

**Key questions**

**What is already known?**

- Between 25% and 40% of those living with HIV and on antiretroviral therapy (ART) in Sub-Saharan Africa stop treatment within 2 years of initiation.
- Clinic location, distance and wait times are well-established reasons for disengaging from care.

**What are the new findings?**

- Inflexible HIV service models that emphasise rigid application of one-size-fits-all clinical monitoring place Zambian patients and providers under pressure.
- Patient–provider relationships are strained by poor working conditions, and disrespect and abuse in clinic settings.
- Zambians in rural and urban areas in this study nonetheless preferred ART for HIV treatment over other alternatives.

**What do the new findings imply?**

- Failures resulting from both health system ‘hardware’ and ‘software’ suggest the need to redesign HIV service models to encourage health worker autonomy and responsiveness to individual patients’ needs.

## Introduction

Between 25% and 40% of the estimated 12 million people living with HIV and on antiretroviral therapy (ART) in Sub-Saharan Africa (SSA)^1^ stop treatment within 2 years of initiation.^2–4^ Engagement in HIV care and treatment (henceforth ‘care’) is necessary to achieve optimal clinical outcomes^5 6^ as it allows for timely and appropriate medication refills, reduction of HIV viral load, monitoring of medication toxicities and, in cases of treatment failure, switching of ART regimens. Engagement in care further facilitates linkage to ancillary services such as prevention of HIV vertical transmission, information services and social support that can help manage HIV.^7–10^ Leaving or ‘dis-engaging’ from routine care puts patients at risk of onward transmission, opportunistic infections and emergence of drug resistance, which reduce drug options and ultimately increase HIV-related mortality.^11 12^

Despite the availability of free ART, patients disengage from HIV care for reasons that vary by setting.^7^ Most of the reported reasons for disengagement from HIV care in SSA have been classified as structural, health facility-related and sociocultural.^7 13^ Examples of structural barriers include poverty, distances and transportation constraints.^14–16^ Some commonly reported health facility-related barriers are frequency of visits needed for ART refills, inefficient service delivery, fear of accidental disclosure, difficult patient–provider interactions, insufficient information about HIV, costs of required medical tests and insufficient healthcare workers (HCWs).^13 17–21^ Reported sociocultural barriers include clients’ preference for alternative HIV medicine, family responsibilities, lack of social support and stigma.^22–29^

In Zambia, an estimated 1.2 million people live with HIV, of whom more than 770 000 are enrolled in HIV care.^30^ Consistent with findings from SSA, Zambia experiences substantial loss to follow-up among patients enrolled in HIV care.^2 4 31^ Four studies conducted in Zambia between 2006 and 2011 found that the use of complementary medicine, lack of trust in Antiretroviral drugs (ARVs), faith healing, side effects and food insecurity all influenced disengagement.^13 32–34^ In one urban settlement in the capital city Lusaka, treatment fatigue, stigma, waiting times, placing ‘defaulters’ on intensive adherence counselling, competing livelihood priorities and dissatisfaction with HCWs’ responses perpetuated disengagement.^13^

This paper presents current data (circa 2015) on patient experiences and responses in the rapidly evolving Zambian HIV care setting with a particular focus on health system factors. Based on emerging themes, we used the constructs of health system ‘hardware’ and ‘software’ to distinguish the impact of structural issues (including resourcing, the role of clinical guidelines and policy—ie, ‘hardware’) from the less tangible but still fundamental issues relating to provider attitudes and work practices (ie, ‘software’).^35^ By focusing on both aspects, we provide qualitative insight into the way quality of care within the Zambian health system contributes to patient disengagement decisions. This insight is critical for informing decisions to reform or adapt current service models to promote greater responsiveness to patients’ varying circumstances throughout a lifetime use of ARV. Although variable,^36^ for purposes of this study, we defined disengagement as the complete set of decisions and actions through which missed clinic visits and ensuing reluctance to return over time erode patients’ subjective sense of connectedness to care.^37^ Analysis more specifically considering the manner in which various individual, social and health system factors *interact* to influence engagement decisions has been previously published.^38^

## Methods

### Research setting

This qualitative study was conducted in 2015 and was nested within a larger quantitative study exploring rates and reasons for disengagement.^39^ It aimed to understand, from Zambian patients’ and HCWs’ perspectives, how care within the health system influenced engagement and disengagement from long-term HIV care and treatment. In our framework, the health system includes the formal system, as well as the patients and the larger community. The qualitative study was conducted in 12 clinics, selected from 4 Zambian provinces—Lusaka, Southern, Eastern and Western provinces. These settings comprise multilingual ethnic groups, with Bemba and Nyanja the most widely spoken local languages in Lusaka Province, Tonga in Southern Province, chi-Nyanga in Eastern Province and Lozi in Western Province. The socioeconomic status and housing conditions of residents in these different locations are mixed, but predominantly poor.

### Site selection

Site selection was achieved in three phases. First, a random sample of 31 health facilities stratified to ensure rural and urban facilities from each of the four provinces were selected. Second, from this larger sample, eight health centres—one urban, and one rural for each of the four provinces—were purposively selected based on facility characteristics, location and patient load. We considered urban health centres and level 1 hospitals interchangeable for the purpose of the qualitative study since they are often of similar size and operating capacity and are located in more comparable socioeconomic and geographical environments. After completing the first round of data collection at these eight facilities, an additional four facilities (one per province) were purposively selected to conduct follow-up focus group discussions (FGDs) that tested the initial findings and probed emerging and unclear issues. Selection was made based on the nature of the issues we sought clarification about—for example, in two provinces rural facilities were selected as clarification and testing of findings related predominantly to these aspects; in the other two provinces, urban facilities were selected.

The 12 clinics all provided outpatient health services, and all the urban facilities additionally provided some inpatient services. Other services shared by the 12 facilities included maternal and child health department (MCH), tuberculosis treatment department (TB corner), HIV or antiretroviral department (ART clinic), and laboratory and environmental health team (EHT) depending on location and resourcing.

The professional HCWs working in the clinics predominantly included clinical officers, nurses, pharmacy technologists, data associates, and environmental and health technologists. In the ART department, the professional HCWs provided counselling, testing, clinical consultations and drug distribution. By comparison, lay HCWs were responsible for HIV testing and counselling, health education, patient navigation and file retrieval, but may also sometimes substitute for professional HCWs and carry out designated clinical tasks such as blood pressure measurements.

### Design and participants

We conducted 69 indepth interviews (IDIs) with patients from the following categories: (1) currently in HIV care at the clinic where they initiated HIV care (‘in care’); (2) disengaged from care; (3) in HIV care after transfer to a different clinic; and (4) next of kin for deceased patients. Among the in care, transferred, disengaged and dead categories, male and female representation was almost equal (male=29/female=31). Geographical representation was also similar, although with slightly higher participation among rural residents (n=38, 55%) (table 1).

**Table 1:** Sex and location of interview and FGD participants

Characteristics	In care	Pregnant (in care)	Transferred	Disengaged	Dead (NoK)	Total (N=69)
	(n=19)	(n=9)	(n=12)	(n=15)	(n=14)	
Gender						
Male	9	–	7	6	7	29
Female	10	9	5	9	7	40
Location						
Urban	7	4	5	8	7	31
Rural	12	5	7	7	7	38

FGD, focus group discussion; NoK, next of kin.

Twenty-four FGDs with a total of 158 participants were conducted with urban and rural lay and professional HCWs to understand their perceived role in patients’ care engagement decisions (table 2). FGDs were separated between lay and professional health workers but were mixed sex.

**Table 2:** Number of FGD participants by cadre and location

Participant type	Urban		Rural		Total	
	FGDs (n)	Total participants	FGDs (n)	Total participants	FGDs (n)	Total participants
Lay HCWs	6	46	6	42	12	88
Professional HCWs	6	38	6	32	12	70
Total	12	84	12	74	24	158

FGD, focus group discussion; HCW, healthcare worker.

Direct observations at health facilities were undertaken to clarify the operational context of care.

### Patient sampling and data collection

#### Indepth interviews

The ‘in-care’ patients were recruited from the files of patients present on a study-visit day using a simple random sampling method. The categories of ‘lost’ patients were recruited during the study tracing exercise with the help of peer educators who were engaged as data collectors of patients lost from HIV care. From each of the categories of ‘lost’ patient (disengaged, transferred and next of kin to dead) traced by the main study, research assistants (RAs) asked participants if they would be willing to take part in a follow-up interview until two participants were recruited from each facility. A balance between male and female participants was sought, although due to pragmatic considerations not always achieved. No patient sampled from the ‘in-care’ patients refused to participate. No patient sampled from the ‘lost’ traced category declined to participate in the interviews after full information was provided by the tracers.

Four Zambian RAs with competence in local languages spoken in the study sites were recruited to collect data. Their recruitment considered previous experience in health-related research. They underwent a 5-day training covering human subjects’ protection, familiarisation with the study’s aim and the study tools, and best-practice approaches to qualitative research data collection. The IDI questionnaire guide was designed in English and translated into the four main local languages used in the study sites, namely Nyanja, Lozi, Bemba and Tonga. IDIs lasted between 40 and 120 min and were conducted in the participant’s choice of language.

We asked patients about their personal experiences while accessing care. We included questions on caregiver attitudes, information availability and sociocultural aspects, and how they affected the interviewee’s perceptions and choices in seeking care. Interview questions were all open-ended to enable RAs to probe for causal mechanisms influencing patients’ engagement in care. The RAs took down summarised field notes that included any non-verbal expressions they observed.

For the deceased, we interviewed a close family member or friend to understand both the sequence of events that led up to death, as well as the families’ perception of the dead patient’s experiences in care and their own role in this process.

#### Focus group discussions

In each of the 12 facilities, the recruitment of FGD participants was achieved by issuing open invitations to all HCWs at the facility to attend one of two FGD sessions. Participants were then enrolled on a first come first served basis. The FGDs for lay and professional staff were separated to enable lay staff to speak freely without the fear of interference from their supervisors.

The FGD guide explored the patient–HCW interaction to understand the relationship between HCWs’ perceptions and patients’ own description of experiences accessing care. The questions were open-ended to enable the facilitator to probe emerging themes, and field notes containing contextual details and non-verbal observations were taken by the RAs to aid in the interpretation and analysis of the data.

FGDs took between 1 hour and 3.5 hours. The main language used in the FGDs with professional HCWs was English, but facilitators allowed the use of local language in discussions. All the FGDs were conducted at the health facilities.

#### Observation procedures

The RAs documented direct observations of healthcare facilities’ operations as field notes that were then formalised into research memos. Direct observations took place in the original eight health facilities for two consecutive ART clinic days and lasted between 8 and 10 hours. The process began with the RA introducing themselves to the person in charge of the facility. The RA would then proceed to sit in a certain department (eg, TB corner, laboratory, pharmacy (where one existed) and clinician rooms) and write down their observations on the research guide. The guide included sections on operational features, intraprovider relations, patient–provider relations and their context. Data from observations helped build a picture of typical workflows and human interactions that drive health centre operations and that influence patients’ experience and decisions related to care-seeking.

### Data analysis

Audio recordings, transcribed scripts and observation memos were saved using a unique identification method and saved on password-protected computers. All audio-recorded interviews were transcribed verbatim and simultaneously translated into English (for interviews in local language). A two-stage approach to quality checking was undertaken. In the first stage, the RAs quality-checked their own scripts and in the second stage the first author quality-checked all the scripts. Anonymised transcriptions, observation memos and notes were imported into QSR NVivo. Inductive methods were used to code the data^40 41^ by the first and last author. Coding was an iterative process that categorised related codes and subcodes and stratified them by study sites and participant engagement status for further exploration and interpretation of the findings. Arrangement of themes according to a conceptual framework (the hardware/software model) was subsequently used to help further refine, organise and reflect on the findings. Draft findings and interpretations were circulated for review to all the study investigators and the Study Advisory Committee.

### Ethics and consent to participate

Written informed consent for participating in the study was sought and granted by all the interviewees who agreed to participate in the study. To ensure privacy and confidentiality, the interviews were conducted in a private environment either at a home or health facility with only the RA and the interviewee present. Permission to record interviews was sought from all the study participants.

## Results

Three major themes emerged on the hardware and software of health systems that inhibit or facilitate long-term engagement in HIV care. First, health system hardware barriers related to resourcing, including the physical infrastructure and the number of HCWs posted at health centres, were identified. Second, health system software, clinic operating practices, work norms and work patterns. Third, also related to health system software, the nature of patient–provider relationships was discussed. In relation to these overarching themes, analysis revealed great consistency across lay and professional HCWs’ and patients’ accounts in both rural and urban settings and was further substantiated by facility observations. Consequently, we do not routinely differentiate results by participant type and location in the following sections, except to highlight those few areas where more meaningful differences or nuances were identified.

### Health system hardware

Consistent across both patient and HCW data sets, respondents mentioned many barriers to engagement in long-term care related to health system ‘hardware’. These included travel times and transport costs associated with clinic location relative to the health facility catchment area; long wait times associated with insufficient and often overworked staff; and lack of privacy associated with inadequate infrastructure.

#### Clinic location

A primary structural barrier to care related to health services was distance to the clinic and associated time and cost to accessing care. Unsurprisingly, rural patients emphasised this as more of a problem than those living in urban areas, although even in urban settings challenges were relayed. Distance played a role both in decisions to disengage and decisions to transfer, as narrated below:

*So we both have that virus. But I was the one who looked after my husband. I collected his medicine too. But I was alone taking care of [him] at home. And it was too much for me because we had different dates. That is how I stopped. […] I would have no transport. I would have to walk. I couldn’t manage*. (Rural Female, Disengaged, Lusaka Province)

As noted by the respondent above, distance and cost of attending the health centre of first enrolment were a central factor in some (and more frequently rural) patients’ decisions to disengage or transfer. Nonetheless, distance was a challenge for other reasons, including for patients who actively chose to enrol in health centres outside their residential area to maintain confidentiality, and those who had moved or travelled for family or work reasons and without the means to pay for regular return transport.

*I just didn’t stop for the sake of stopping! But I had encountered a problem. I never had money to travel back and where I was working, they never paid me on time. So, I failed to travel back*. (Rural Female, Disengaged, Western Province)

*I transferred from [my first clinic] because I divorced my husband who lived there. So it became far for me to come from here to go back there [for ART]. That is what made me transfer from there to this mission here*. (Rural Female, Transfer, Southern Province)

#### Clinic infrastructure

Some patients complained of cramped infrastructure leading to constant overcrowding and lack of privacy for basic consultations, conditions confirmed by a number of HCWs and through direct observation.

*[The patients] go to far places […] in the search of confidentiality. It’s not like they don’t want to take that drug. They want help so that they can live better lives. But they are scared […] with the setup here. From that gate, you go straight to the ART. So even when I am at the OPD [outpatient department] I am able to see those people. So they avoid coming here, because they want privacy*. (Lay HCW, Urban Site, Eastern Province)

Although not a central determinant, when combined with reported concerns about involuntary disclosure via community gossip, lack of clinic space or infrastructure to assure privacy emerged as a potentially important barrier to accessing or maintaining long-term engagement in care, as stated below:

*When an individual goes to the clinic, there are people […] They will come to see what goes on there at the ART clinic so they can come and tell people what is happening. And that is what makes me feels lazy [to go for care* *]*. (Rural Female, Disengaged, Eastern Province)

#### Staff shortages

Insufficient HCWs to handle the high numbers of enrolled patients with HIV was a common frustration expressed by providers and patients in both rural and urban sites in this study.

*We face a lot of challenges. One, we have not enough man power which is a cry of each and every clinic in the country*. (Professional HCW, Rural Site, Eastern Province)

*The way I have observed things at the clinic […] Maybe if they can just increase the number of doctors so that queues can be reduced. Because there are long queues like from here to that mango tree to be attended to by one doctor. But if they can add another doctor, the queue can be shortened*. (Urban Male 1, Disengaged, Western Province)

As noted above, many respondents saw lack of staff as a critical driver of long queues and waiting times. While some patients were prosaic about this reality, others described these factors as influencing their decisions to transfer, as described below:

*I transferred because […] my husband said it’s just too congested. We tried to go there the other day. It was congested. There were a lot of people. We waited and we got tired and he said: “No! This place is not conducive for us! Maybe, let’s go [elsewhere].”* (Urban Female, Transfer, Lusaka Province)

### Health system software

Emerging themes relating to health system software are broadly grouped under ‘Discretionary workplace practices’—defined as day-to-day operational and clinical decisions that lie within the control of front-line health workers—and ‘Health Worker Attitudes’.

#### Discretionary practices: clinic opening hours

Clinic hours and particularly opening times were frequently mentioned as frustrating patients’ access to care. The degree to which late opening or early closing times acted as a disincentive varied between patients. Those registered in rural facilities were most materially affected, reporting that they travelled greater distances only to meet clinic bottlenecks due to late opening times.

*When it comes to opening the clinic, they are usually late. They can open at 08:00 but for them to start working, they usually delay. You will find that they will start at 09:00 or 10:00 and by then you will be already tired of waiting. So that makes it hard*. (Rural Female, In Care, Eastern Province)

In FGDs, professional HCWs revealed that there was a strong informal or ‘practical’ norm of late opening and closing early in many health centres. Health workers justified these known breaches of formal Ministry of Health (MoH) guidelines in various ways:

*Here, [HIV care] is every day. But mostly we concentrate in the morning from 08:00 to 13:00 before lunch. Because after lunch, most of our adherence counsellors will have knocked-off and they are the ones who help us in pulling the files*. (Professional HCW, Rural Site, Eastern Province)

#### Discretionary practices: file and record management

Patients’ motivation to stay in care was negatively influenced by poor management of files and medical records, which contributed to long waiting times through long and sometimes fruitless searches for paperwork:

*The way they keep the files, the file was lost daily. When I came, each and every time, the file was lost! They’ll say: “We have not seen it.” And that’s that!* (Urban Male, Transfer, Lusaka Province)

Matching direct observations, some patients reported attending health centres where lost results or files meant they had to redo laboratory tests, requiring additional visits and more waiting. While it was never provided as a stand-alone reason, poor record-keeping was often a contributing factor to decisions to transfer or disengage:

*I was at the lab. After two weeks they told me to come back [for the results] but the file was lost, you see! But how did it get lost! So all those things [influenced my disengaging]*. (Urban Female 2, Disengaged, Lusaka Province)

HCWs generally acknowledged and attributed poor file management to work overload, reduced support from non-governmental organisations, lack of physical storage space and increasing patient numbers.

#### Discretionary practices: drug rationing and inflexible visit schedules

No respondents in this study reported experiencing a complete ARV stock-out. However, a subset of respondents in all study sites described HCWs as setting apparently arbitrary limits, such as 1 months’ worth of medication refill when national guidelines allow for 3 months for stable adherent clients. Others questioned the relevance of the 3-month limit when clinical assessment of immunological or virological status occurred every half year. In almost all cases, having to make frequent returns to the clinic incurred time and financial costs and interfered with work or family commitments.

*Because they give three months [of drugs] but you have been taking the medicine for three years. So you find that you are knowledgeable and are in line with the medicine without any reactions. So at least if they would put/give for six months so that we can stay for a long period, it would help*. (Urban Male, In Care, Southern Province)

*Like I have explained, I am a bricklayer. Once or even three times I told them: “I am going to work and that I don’t know when I can come back. So that one month [of drugs] you are giving me its better you give me for 3 months.” But they refused saying: “It’s our program, so it’s better we give you something for 1 month.” So I tend to think that these people have not thought about me*. (Rural Male, Transfer, Eastern Province)

Independent of the issue of medication refills, other patients noted the punitive nature of ‘intensive adherence counselling’ for patients who had missed visits. As per national guidelines, HCWs often (although not always) required patients who missed visits to return weekly or fortnightly for regular adherence counselling. For some patients, such rules meant a continual clash and ultimately a trade-off between meeting basic livelihood and treatment requirements.

*I stopped going there* [the clinic] *because they gave me a lot of [scheduled visit] days […] So I thought that let me just stop*. (Urban Female, Disengaged, Western Province)

Providers in all four provinces described invoking these rules (with some small variations) to impress on patients the need to take their drugs on time, as illustrated by the quote below:

*When a patient has missed an appointment by a number of days, we – it’s not a punishment in the real sense – but, we punish the client when they come back. Instead of giving such a patient drugs that would last for maybe three months, we give them drugs to last two weeks so they can get used to remembering frequently that they have to come to the clinic*. (Lay HCW, Rural Site, Eastern Province)

Although lay health workers were often responsible for adherence counselling, this cadre report to and were encouraged by professional health workers to invoke ‘intensive visits’, with an emphasis on ensuring routine clinic attendance to facilitate clinical monitoring and health education, as noted by these professionals:

*Most of the time you find that the client might not be happy with that [intensive adherence] […] but with explanation on the importance of the counselling and the treatment, it is like you just re-emphasise on the importance of the treatment*. (Professional HCW, Urban Site, Southern Province)

*It’s something to do with compliance. The patient just has to follow what they have told them. It’s still the same thing whether we are discussing the food, drugs, how to live a health life. It comes back to, are they following what we are telling them? That’s the adherence*. (Professional HCW, Rural Site, Southern Province)

#### Alternative treatments

Although many patients complained over access to, and quality of care in, formal health facilities, these services were nonetheless compared favourably with other, alternative treatments by the majority of our study participants. While a few individuals reported having sought help from an alternative provider at some stage, most said that they had never sought or used traditional medicines. Three main reasons for not seeking alternative care were reported.

First, patients thought that health centre processes were more rigorous than those used by traditional healers. Health centre’s use of equipment (eg, blood pressure cuffs, thermometers) and blood tests to establish baselines and monitor ongoing health status, along with provision of health education and information, were all contrasted with traditional practice.

*It is different [with a traditional healer] because that one won’t explain to you what is paining you, or how it will work with the medicine he will give you*. (Rural Female 2, Transfer, Southern Province)

Second, an important new finding from this study was that many respondents believed that the care provided at the MoH clinics was superior and preferable to that by alternative health practitioners. Participants frequently noted that traditional medicines, unlike ARVs, were not ‘strong enough’ for HIV even if they ‘might have worked in the past’ or may still work for other conditions.

*These [ARVs] are working. I have not gone to another place for a traditional healer because that medication doesn’t do anything. As for the traditional medication, it doesn’t work nowadays. [Although in] those old days, the traditional medication used to be very effective*. (Urban Female, In Care, Eastern Province)

Finally, many patients commented that treatment at the health centre was free, while traditional healers just ‘took your money’ without delivering promised results.

*Mmmmmm the traditional healer can only finish your money. But at the clinic there’s nothing paid, it’s free of charge*. (Urban Male 1, Disengaged, Western Province)

#### Health worker attitudes

While presented last, HCW attitudes and respect formed a central pillar of many patients’ accounts about long-term engagement and disengagement in care.

#### Health workers professionalism

Most respondents said that HCWs at their clinic were the most likely source of trusted information about HIV care. Respondents, even some disengaged patients, emphasised that HCWs often improved their understanding and ability to cope with their diagnosis and treatment by providing ‘education’ and helping ‘teach’ patients about their disease.

*The benefit is there because I have learnt a lot from [the staff]. The first thing they taught us was how to live, how to eat and how to keep ourselves*. (Urban Female, Transfer 2, Lusaka Province)

Despite such acknowledgement, many patients also described feeling demotivated to seek or remain in care due to HCWs’ weak professionalism, which, while not attributed to all, was described by a subset of patients at all study sites.

Concerns primarily focused on timeliness, attention to detail, efficiency and confidentiality. With regard to timeliness, a common complaint was that HCWs were lazy or lax—they ‘chatted’ or ‘gossiped’ or took unnecessarily long tea breaks while patients were waiting. ‘Poor attitude’ or lack of work ethic among HCWs was described in relation to a range of factors including variable opening times, poor filing (and loss of files) and general lack of efficiency, as outlined below:

*Just the way they do things, that is our big complaint! Even when the doctor comes, I will tell the doctor: “These people if they cannot manage those jobs, we should help, because we can manage putting the files in order so that whoever comes, we know where the things are!”* (Rural Male, Transfer, Western Province)

*But when you try to explain some things, they say you are rude and whatever. But […] they make mistakes too*. (Urban Male 1, In Care, Lusaka Province)

Respondents also complained of HCWs prioritising or ‘fast tracking’ their own family and friends for treatment. The extra waiting time and difficulty in garnering information or attention without such connection, whether coincidental or deliberate, were factors that contributed to some patients’ decisions to leave care.

*Just here, okay one thing that I have seen here is that ah [reception at the clinic] is difficult. It is very difficult the way you are received, if you do not know anyone here, then Ah! Things will not move well!* (Urban Female 1, Disengaged, Lusaka Province)

HCW confidentiality—or lack thereof—was another recurring theme related to professionalism and raised by both patients and even some HCWs as damaging patient–provider relations and acting as a disincentive to care:

*I have observed something which goes wrong that affects the clients. There is that tendency of disclosing the status to the relative minus the consent of the client […] It doesn’t happen frequently but I have observed a number of times by myself, maybe even I have done it. But it is another thing that put clients off, because when we start dealing with them, we vow to them that we are going to keep the information confidential*. (Lay HCW 4, Rural Site, Eastern Province)

#### Health workers’ service values and respect

Finally, in all clinics across the four study provinces, patients reported encountering HCWs who were disrespectful or abusive. Patients narrated experiencing bullying behaviour, being shouted at and publicly humiliated particularly in the event of ‘late’ arrival for an appointment at the clinic or in response to requests that professional HCWs perceived to be unreasonable. Such experiences were several times linked to patient decisions to stay or leave the clinic.

I: Did you feel like the health care workers cared about you?

*R: Ah! They care rudely! They don’t care as you are taking care of me right now, if at all! I wish it was you who was there - they care rudely!* (Urban Male 1, In Care, Lusaka Province)

*When you go [to the clinic] and ask [questions], they would shout at you. But they are not supposed to shout at us. Instead they are supposed to encourage that person […] But just to say the truth, one of the reasons why I stopped care is because there they shout at us very much - They are rude*! (Urban Female, Disengaged, Eastern Province)

Even though patients appreciated and understand that HCWs were helping them with their treatment, they were also aware that they are being mistreated.

## Discussion

Our indepth exploration of health system factors provides a more complete and nuanced understanding of current perceptions and experiences of Zambians living with HIV that contribute to engagement and disengagement from long-term care. The findings were remarkably consistent across the rural and urban participants, with variations mostly in the extent to which certain factors hindered or facilitated engagement in care. The health system ‘hardware’ (resourcing) and ‘software’ (clinic operating practices—including work norms and patterns and HCW attitudes) often interacted and amalgamated to influence patients’ decisions to engage or disengage in care.

This paper presents evidence that Zambians in both rural and urban areas currently perceive ART and MoH services as being of better quality and more efficacious than alternative, traditional treatments. This finding is contrary to previous studies in Zambia which reported preference for alternative treatment in many settings due to ART-induced side effects, dissatisfaction with ART care, inability to meet stringent ART regimens and the quest to ‘get cured’.^13 31–33^ The preference for MoH services expressed by Zambians in this study was partly due to respondents’ perception that medical diagnostic tests and results provided the basis for more reliable and targeted treatment, perceived efficacy of ARV drugs and the provision of ARVs free of cost. This emergent preference shows an encouraging evolution in Zambians’ awareness of, and confidence in, government-provided ART treatment at the individual level. It suggests that Zambians living with HIV may be motivated to both try and stay on ART due to changes in perceived benefits of taking ART.^42 43^ However, our findings also show that improving patient experiences within the health system would help patients maintain ART use and keep them engaged in care.

The substantial impact of clinic location and distance to travel on access to care is well documented in both Zambian and regional literature.^15 28 44–46^ This study reaffirms the need to make HIV care more easily accessible, especially in rural areas. In part because of ongoing geographical barriers to access, our findings also highlight the need for HCWs to better understand and respond to individual patients’ situation with flexibility. For example, where possible, by giving a longer supply of drugs if a patient cannot return for frequent appointments. HCWs that play an empathetic supportive role can reduce their social distance from patients, making patients more free and open about their experience with HIV care, which in turn may improve patients’ comprehension of received advice and willingness to remain engaged.^47–49^

Additionally, decentralised and ‘differentiated’ approaches urgently need to be explored as a mechanisms for accounting for patients’ varied socioeconomic circumstances.^50–53^ For example, community-level approaches that bring home-based HIV testing, ART initiation and HIV services into the community, or allow for family-centred or community-centred care, could be acceptable and feasible ways to address ‘access to care’ challenges.^44 54–57^ Community-level approaches to HIV service delivery could increase HIV awareness and foster critical (but often absent) collective support for those seeking to initiate or remain in HIV care.^44 58–61^ Such a model could help bring HIV care closer to the communities, but also help transform communities into partners in the provision of HIV services, giving them room to participate in adapting the HIV services to patient needs.

The intersecting role of human resource shortages and discretionary practices (eg, late opening hours) discourage some patients—especially those who are more financially or socially vulnerable—from engaging in care. Chronic human resource shortages^62 63^ lead to bottlenecks and long queues that are natural causes of frustration.^64–66^ Also, routine operating practices that include late opening times, weak time management and occasional nepotistic practices contribute to patients’ sense of ‘wasted time’ and also vulnerability to unintended disclosure in the clinic setting. These factors combined (and sometimes additionally influenced by other factors such as fear of losing social and emotional support due to anticipated stigma and insecure labour conditions)^13^ contribute to decisions to transfer or disengage.

Integrating more lay health workers formally into the health system to work at the community level or task-shift in facilities could be one strategy to mitigate staff shortages and frustrations with service delivery.^67 68^ Coming from the patients’ communities may position lay health workers to strengthen patient trust and to contribute considerably to improved health services.^69 70^ Some lay health workers have amassed experience which is cardinal to meet the acute shortages of HCWs in health facilities.

However, simple addition of more health workers or increased training alone is unlikely to resolve the issues highlighted in this study. Socialisation into the workplace, governance, management of workload and remuneration all need careful consideration, else lay health workers may not be able to function and enhance service responsiveness as intended.^71–73^ A study in Kenya, for example, reported concerns with the professional conduct and power relations between lay workers and patients due to the former being powerful members of their communities and acting as gate keepers.^74^ Selection of appropriate providers requires careful consideration of those characteristics most likely to imbue trust, and support, training and supervision are all critical to ensure quality and responsiveness are maintained over time.^75 76^

Many patients described the quality of patient–provider relationships as a factor contributing to both engagement and disengagement from HIV care. This finding is consistent with other studies where disengaged participants expressed a disconnect between their expectations of care and the providers’ style of care delivery.^71 72 77 78^ As demonstrated in the literature, HCWs, particularly professional HCWs, inevitably command a position of power in the healthcare setting.^21 71 73^ Thoughtless reprimands designed to highlight this power differential creates a stressful environment for patients, and both in this study and elsewhere have been documented as discouraging clinic visits.^13 21^

The critical and supportive role that empathetic providers play in supporting some participants showcases the importance of providers’ responsiveness to engagement in care. Studies have shown that positive relationships with HCWs increase patients’ confidence in their recommendations and motivate them to remain in HIV care.^14 46–48^ However, HCWs need support to play this empathetic role through adequate resource allocation, effective leadership and management,^79^ incentives, and continuous training and mentoring opportunities.^46 80^

In this study, both lay and professional HCWs acknowledged that putting patients on intensive clinic visits if they failed to keep to clinic appointments was viewed by patients as a ‘punishment’ and did not address the constraints that led to such ‘failures’. In this regard, there is a need to better understand variable responses by HCWs to patients’ needs, for example, why do some demonstrate a willingness to be ‘flexible’ in their application of the rules (in ways that generally facilitate engagement) while others demonstrate great rigidity? What is it really about patient–provider relationships that facilitates or inhibits retention and adherence?

### Study limitations

This paper reports findings from a qualitative study. Thus, while we collected data from a large number of patients and health workers taking care to ensure proportionate male and female, lay and professional, and urban and rural representation, our findings are not directly generalisable. We also recognise that the extended contact and engagement with participants may have led them to overstate or understate the circumstances leading to their engagement decisions, or to health workers emphasising certain aspects of their care over others. To minimise this risk, we used audio recordings and field notes to evaluate non-verbal communication. We also triangulated interview data against those generated from direct (non-participant) observation. While specific findings may not be generalised beyond the four Zambian provinces, we believe our analysis is an accurate reflection of participants’ experience and sense-making of their treatment within the Zambian social and health system setting.

## Conclusion

This paper suggests that people living with HIV in our sample prefer ART over other alternative HIV treatments, yet they may disengage from long-term care partially due to health system ‘hardware’ and ‘software’ factors. Insufficient resourcing in terms of clinic location, infrastructure and staffing, along with ‘discretionary workplace practices’, seemingly arbitrary clinical decisions, and disregard or abuse by some front-line HCWs, led patients to disengage and were perceived by all respondent types as factors leading to disengagement from ART clinics in Zambia. We therefore suggest a need for improvements in resourcing and structuring of HIV services and reorientation of healthcare policy, starting from preservice training to clinic operational guidelines and clinic leadership, to encourage more ‘patient centred’ care. ART delivery must be in consultation with the ART recipient communities, which could create an improved and more equal patient–provider relationship. The ensuing sense of shared responsibility in delivering ART that is suited to patients’ needs and context could keep patients engaged in long-term care and help achieve desired health outcomes.
